# Pulmonary Edema Occurring after Nitric Acid Exposure

**DOI:** 10.1155/2019/9303170

**Published:** 2019-01-15

**Authors:** Christopher W. Meaden, John S. Kashani, Stephen Vetrano

**Affiliations:** ^1^Department of Emergency Medicine, St. Joseph's University Medical Center, Paterson, New Jersey, USA; ^2^Department of Emergency Medicine, Capital Health System, Trenton, New Jersey, USA

## Abstract

Nitric acid (HNO_3_) is a strong acid and oxidizing agent used for various applications including production of ammonium nitrate in the fertilizer industry. Nitrogen oxides formed when nitric acid interacts with the environment have been implicated in inhalation injuries. This describes a case of a 49-year-old male who presented to the emergency department complaining of an acute onset of shortness of breath approximately 12 hours after being exposed to nitric acid fumes. He presented with a room air oxygen saturation of 80 percent with moderate to severe respiratory distress. His plain film chest radiograph showed bilateral pulmonary infiltrates and pulmonary edema. Over a seven-day hospital course, he had an improvement in his clinical status and chest X-ray with normal pulmonary function tests one month after discharge. Although exposure to the fumes of nitric acid is known to cause delayed pulmonary edema, it is rarely reported in the medical literature. This case serves as a reminder to consider exposure to fumes of nitric acid in a patient presenting with pulmonary edema and highlights the importance of obtaining a work history.

## 1. Introduction

Nitric acid (HNO_3_) is a strong acid and oxidizing agent and is used for various applications, with one of its main uses being the production of ammonium nitrate in the fertilizer industry as well as other industrial applications. Its ability to nitrate organic compounds makes it an ideal agent for this purpose. Pure nitric acid is a colorless liquid that boils at 84.1°C and can undergo partial decomposition to form nitrogen dioxide (NO_2_). The nitrogen dioxide will impart a yellowish discoloration to nitric acid; at higher temperatures a red discoloration is appreciated. Pure nitric acid tends to give off white fumes when exposed to air while nitric acid with nitrogen dioxide admixed will give off reddish-brown vapors [1–3].

The application of nitric acid will also generate various oxides of nitrogen including nitric oxide (NO), dinitrogen trioxide (N_2_O_3_), dinitrogen tetroxide (N_2_O_4_), and dinitrogen pentoxide (N_2_O_5_). These chemicals are easily interconverted under various conditions. Of the various nitrogen oxides, nitrogen dioxide is the most important regarding human exposure. Nitrogen dioxide is a sweet smelling red-brown gas that is denser than air. Nitrogen dioxide tends to collect at the bottom of enclosed spaces. It has limited water solubility and therefore is not irritating to mucous membrane and the upper respiratory tract allowing for a prolonged exposure, which can cause a chemical pneumonitis, from an unrecognized significant exposure, up to 24 hours after exposure [1–3].

Inhalation injury from nitric acid, as well as its oxidized derivatives, has been shown to cause local tissue inflammation within the lower respiratory tract leading to symptoms. The most common exposure to nitric acid is chemical burns causing a yellow discoloration of the skin; however, this manuscript discusses a case of pulmonary complications. Clinically, nitric acid inhalation injury severity has been linked to duration and amount of gas exposure. Typically, exposure has been described beginning with mild upper respiratory irritation. A latent period has then been described which will last anywhere from 3-24 hours ending with the development of symptoms of pulmonary edema and can develop into respiratory failure [1–3]. Here, we report the case of a 49-year-old male working with nitric acid that developed pulmonary edema 12 hours after being exposed.

## 2. Case Report

A 49-year-old male nonsmoker, with no past medical history, was working with nitric acid in an enclosed area. Upon noticing a reddish-brown sweet smelling gas emanating from the bottom of a 55-gallon drum, he turned on exhaust fans but continued to work. He did not put on any kind of protective mask or respirator on. He felt the sensation of eye and throat irritation and shortness of breath. During the course of the six-hour exposure, he, on multiple occasions, retreated to the outside area and felt an amelioration of symptoms. Approximately 12 hours later he experienced paroxysms of cough and shortness of breath and was driven to the emergency department by his wife.

He presented to the emergency department in moderate to severe respiratory distress. Physical examination revealed an oral temperature of 98 degrees Fahrenheit, respiratory rate of 34 breaths per minute, blood pressure of 118/61 mm/Hg, and pulse of 87 beats per minute, and room air oxygen saturation was 80 percent. There were no murmurs rubs or gallops. Diminished breath sounds were appreciated on lung examination. There were frequent paroxysms of cough which were exacerbated by deep inhalation; there was no use of extra inspiratory muscles and no cyanosis appreciated. The remainder of the exam was normal. He was placed on supplemental oxygen at 2 liters per minute with an increase in his oxygen saturation to 85 percent. The supplemental oxygen was increased to 4 liters per minute with an increase in his oxygen saturation to 92 percent and he was given bronchodilator treatments.

**Figure 1:**
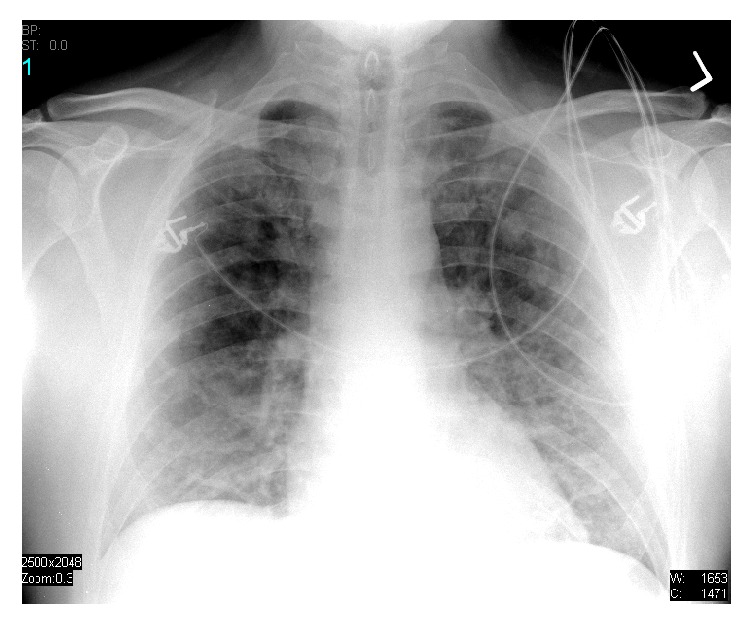
Initial chest X-ray.

On 2 liters of supplemental oxygen by nasal cannula, his arterial blood gas showed a pH of 7.37, pCO2 44.4 mmHg, pO2 44.1 mmHg, and bicarbonate 25.3 mmol/L, and base deficit was 0.2 mmol/L. Carboxyhemoglobin and methemoglobin levels were unappreciable. Normal blood gas values are pH of 7.36 – 7.44, pO_2_ of 80–100 mmHg, pCO_2_ of 36–44 mmHg, and HCO_3_^−^ of 22–26 mmol/L. All other laboratory values were within normal limits with the exception of a white blood cell count of 12.9 k/mm^3^ (normal range: 4.5 to 11.0 k/mm^3^). Chest radiography showed bilateral pulmonary infiltrates and pulmonary edema (Figure 1). Electrocardiogram showed normal sinus rhythm with a ventricular rate of 87 beats per minute with a normal axis and normal intervals.

**Figure 2:**
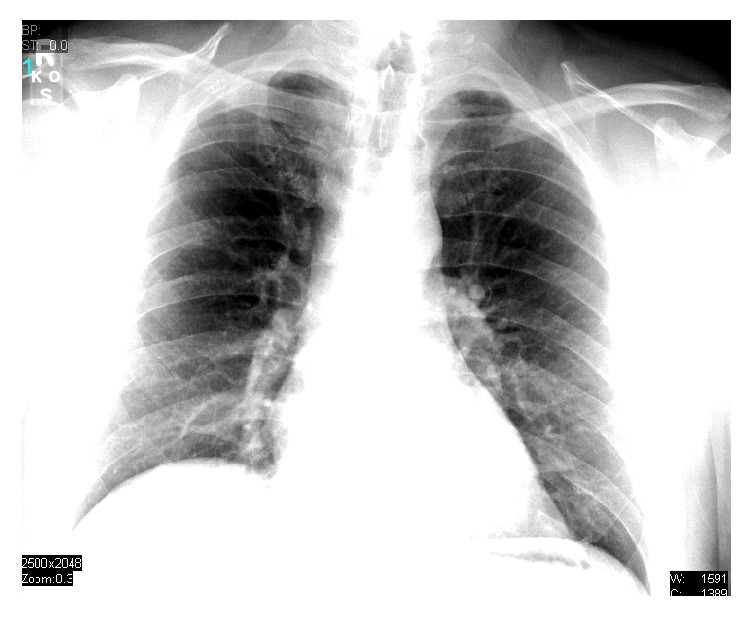
Chest X-ray seven days after admission.

He was admitted to the intensive care unit, supplemental oxygen was continued, and bronchodilator treatments using albuterol and ipratropium (2.5 mg and 0.5 mg, respectively) were given every six hours. Pulmonary care for this patient was at the discretion of the pulmonologist on the case. Over a seven-day hospital course he had progressive improvement in his symptoms and his chest X-ray (Figure 2). One month after discharge the patient presented to pulmonary re-evaluation and followup. At the time of outpatient followup the patient denied any complaints at that time. During the visit standard pulmonary function testing was performed which included forced vital capacity, forced expiratory volume in the first second, peak expiratory flow, and maximum mid-expiratory flow. These pulmonary function tests were found to be within their normal parameters. After this visit, he was lost to follow up.

## 3. Discussion

Oxides of nitrogen are emitted from a variety of sources. Of the nitrogen oxides, nitrogen dioxide is the most important with regard to human exposure. The major contribution of atmospheric nitrogen dioxide is from the combustion of fossil fuels and emissions from motor vehicles [4]. Indoor exposure of nitrogen dioxide can occur through the use of gas stoves and heaters, including kerosene space heaters. Additionally, cigarette smoke can be a source of nitrogen dioxide.

Potential exposure to nitrogen oxides can occur in various occupational settings. Electroplaters, acetylene welders, nitrocellulose combustion, explosive detonation, and the application of nitric acid are all potential occupational exposure risks. In the agricultural setting the generation of nitrogen dioxide can occur from the decomposition of organic materials (corn, grains) that are deposited in silos [5]. The exposure to nitrogen oxides in this setting has been termed “silo fillers disease.. Ice skating rink resurfacing machines (Zamboni) have the potential to generate nitrogen dioxide resulting in lung injury [6, 7]. Cold blast furnace syndrome, resulting from exposure to nitrogen oxides, has also been described [8]. The magnitude of exposure, duration, and comorbidities determine the severity of illness after nitrogen dioxide exposure.

Although specific mechanisms leading to lung injury are not fully elucidated, it is thought to occur by a combination of free radical injury, nitrogen dioxide generation of nitric acid with mucosal membrane contact, a decrease in *α*-1-protease inhibitor, lipid peroxidation, thiol oxidation, and the formation of 3-nitortyrosine [9]. These deleterious effects that lead to lung injury affect both type I and type II alveolar cells.

Throughout the literature, symptoms of nitric acid inhalational injury have been generalized into three phases—acute, subacute, and delayed onset. The acute exposure leads to an immediate onset of chest pain, wheezing, shortness of breath, cough or palpitations, nausea and vomiting, and diaphoresis; sudden death secondary to laryngospasm and bronchospasm has been described as well. Patients who present with subacute disease have nonspecific symptoms that can include dyspnea, cough, generalized weakness, and nausea; the symptoms can persist for up to 2 weeks but rarely persist for that length of time. Those patients, who present with delayed symptoms, as with our patient, typically present with onset of symptoms within 4 to 12 hours after exposure; the delayed symptoms include dyspnea, tachypnea, cyanosis, bronchospasm, hemoptysis, tachycardia, and chest pain. Delayed symptoms are caused secondary to the development of frank pulmonary edema and progression of the patient into acute respiratory distress syndrome (ARDS) [1].

There have been very few cases related to nitric acid inhalational injury documented in the literature; even rarer than reports of pulmonary edema from nitric acid inhalational injury are reports of mortality. In 2010, Murphy et al. presented a case of a pulmonary edema after acute occupational exposure to the nitric acid which resulted in the death of the patient. In their manuscript, they reviewed at the time, three other case reports that were related to vaporized nitric acid inhalational injury; these cases described both patients who succumbed to respiratory failure and who survived [3].

When patients present with exposure to nitric acid, they should at minimum be observed for 24 hours given the possibility of delayed presentation, as with the patient presented in this manuscript. Symptomatic treatment of lung inhalational injury from HNO_3_ is largely supportive and remains unstandardized. Treatment plans that have been described include respiratory support, corticosteroids, and nitrous oxide (NO). The patient described by Murphy et al. received both NO and corticosteroids, however, did not survive. There is no specific literature that supports efficacy of NO in nitric acid induced pulmonary edema and acute respiratory distress syndrome; however, there are numerous case reports that suggest efficacy of corticosteroid usage in the treatment of patients with HNO_3_ induced lung injury [3, 10].

It is important to fully evaluate patients with exposure to nitrogen dioxide as there are more systemic complications from exposure outside of skin and pulmonary complications. There are reports in the literature of the formation of methemoglobinemia which have been published through skin burns associated with nitrogen free radicals [11]. More recently, there are reports of patients diagnosed with methemoglobinemia after ingestion of amyl nitrate [12, 13]. Although the patient presented in our case had undetectable levels of methemoglobinemia, this is something to consider in patients that present with inhalational exposure and an area in this field that should be further investigated.

In 2017, Kido et al. present a case of nitric acid induced pulmonary injury with improvement after administration of corticosteroids. In their case they note that the patient did not begin to show signs of improvement until time of administration of corticosteroids. In their manuscript they further review prior case reports of HNO_3_ exposure and treatment, noting most received corticosteroid therapy [10]. Further they note that, in one case report by Lee et al. (2013), a patient survived after receiving corticosteroid therapy for only a short 5-day course; however, the patient developed bronchiolitis obliterans, a possible sequela of nitric acid lung injury 1 month later. [14] The authors thus recommend that a longer course of steroids is beneficial in the treatment of pulmonary edema/ARDS from nitric acid inhalational injury and prevention of subsequent sequelae. This evidence is just one case report, and at this time, there remains no decisive consensus on the utilization of corticosteroids in the treatment of acute pneumonitis secondary to chemical inhalation; with that in mind, it has been better elucidated that corticosteroids may be beneficial in the treatment of later onset bronchiolitis obliterans [10, 14].

This case demonstrates the development of pulmonary edema occurring after exposure to nitric acid fumes. Cases of delayed onset pulmonary edema occurring with the application of nitric acid are not commonly reported in the medical literature. The clinician should be aware of the potential development of delayed pulmonary edema after nitric acid exposure.
